# Fibrinogen is an Independent Risk Factor for White Matter Hyperintensities in CADASIL but not in Sporadic Cerebral Small Vessel Disease Patients

**DOI:** 10.14336/AD.2020.1110

**Published:** 2021-06-01

**Authors:** Xingfang Guo, Bin Deng, Lizi Zhong, Fen Xie, Qing Qiu, Xiaobo Wei, Wenya Wang, Jiangping Xu, Ganqiang Liu, Wong Peter Tsun Hon, Midori A. Yenari, Shuzhen Zhu, Qing Wang

**Affiliations:** ^1^Department of Neurology, Zhujiang Hospital of Southern Medical University, Guangdong 510282, China.; ^2^Department of Radiology, Zhujiang Hospital of Southern Medical University, Guangdong 510282, China.; ^3^School of Pharmaceutical Sciences, Southern Medical University, Guangzhou 510515, China.; ^4^School of Medicine, Sun Yat-sen University, Guangzhou, Guangzhou 510515, China.; ^5^Department of Pharmacology, Yong Loo Lin School of Medicine, National University of Singapore, Singapore.; ^6^Department of Neurology, University of California, San Francisco & the San Francisco Veterans Affairs Medical Center, San Francisco, CA, USA.

**Keywords:** fibrinogen, CADASIL, cerebral small vessel disease, white matter hyperintensities

## Abstract

The relationship between fibrinogen and white matter hyperintensities (WMHs) are inconsistent. Whether there are different relationships between WMHs and fibrinogen in disparate subtypes of cerebral small vessel disease (CSVD) remains unknown. Here, we investigated the roles of plasma fibrinogen in sporadic CSVD (sCSVD) and Cerebral Autosomal Dominant Arteriopathy with Subcortical Infarcts and Leukoencephalopathy (CADASIL) patients. We performed a cross-sectional study that included 74 CSVD patients (19 CADASIL and 55 sporadic) and 74 age- and gender-matched healthy controls (HCs). Plasma fibrinogen was determined, and the severity of WMHs in CSVD patients was rated according to Fazekas scales. Univariate analysis and ordinal logistic regression were performed to evaluate the relationship between fibrinogen and the severity of WMHs in CSVD. Both CADASIL and sCSVD patients showed significantly higher plasma fibrinogen levels than HCs. No significant difference in the plasma fibrinogen level was observed between CADASIL and sCSVD. Univariate analysis and ordinal logistic regression indicated that fibrinogen is an independent risk factor for the severity of WMHs in CADASIL patients (odds ratio [OR] =1.064; 95% Confidence interval (CI, 1.004-1.127); p =0.037). However, age (odds ratio [OR] =1.093; 95% CI (1.033-1.156); P = 0.002), but not fibrinogen (odds ratio [OR] =1.004; 95% CI (0.997-1.011); P=0.262), is an independent risk factor for the severity of WMHs in sCSVD patients. Our results suggest that high levels of plasma fibrinogen are associated with the severity of WMHs in CADASIL but not in sCSVD patients, indicating that the role of fibrinogen may be different in disparate subtypes of CSVD. A better understanding of fibrinogen may yield insights into the pathogenesis of CSVD.

Ours and other studies have shown that cerebral small vessel disease (CSVD) is a highly prevalent condition in older adults and a significant contributor to stroke and cognitive impairment [1-8]. Cerebral autosomal dominant arteriopathy with subcortical infarcts and leukoencephalopathy (CADASIL) is a genetic paradigm of CSVD caused by mutations in the NOTCH3 gene; it is characterized by recurrent ischemic events at early or middle adulthood, mood disturbance and, particularly, subcortical dementia [4, 9-13]. One of the hallmarks of CSVD is the presence of white matter hyperintensities (WMHs) on brain magnetic resonance imaging (MRI); the change in blood-brain barrier (BBB) permeability is proposed as a cause of WMHs [14, 15]. An increase in blood-brain barrier (BBB) permeability is an important pathophysiological mechanism in CSVD, and the disruption of its integrity may lead to indiscriminate leakage of circular components such as fibrinogen (an important marker of BBB dysfunction [15]) into the brain and subsequent deteriorated cognition [16, 17].

Fibrinogen, as a marker of BBB leakage due to its relevance to WMHs [14, 15], is a plasma coagulation protein synthesized by hepatocytes circulating in the bloodstream that performs multiple activities in the hemostasis system [18]. After BBB disruption, fibrinogen leaks into the central nervous system (CNS) and activates a series of neuro-inflammatory mediators, scar formation and myelin abnormalities [19]. Therefore, fibrinogen is not only a marker of BBB disruption but also a driver of inflammation and neuropathology. The relationship between fibrinogen and WMHs has been reported in some population-based studies, but the results have been inconsistent [20]. Whether different relationships exist between WMHs and fibrinogen in disparate subtypes of CSVD has been rarely studied. To address those gaps, the current study aimed to assess the association between fibrinogen and the severity of WMHs in different subtypes of CSVD (CADASIL *vs* sporadic CSVD).

## METHODS

### Study design

This study is a cross-sectional analysis of collected data from patients admitted for NOTCH3 genetic testing at Zhujiang Hospital. This study was approved by the ethics committees of the Zhujiang Hospital of Southern Medical University and complied with the principles outlined in the revised Declaration of Helsinki of 1975 and 1999 National Institutes of Health Human Subjects Policies and Guidance. All the participants provided written consent to participate in the investigation and allowed investigators to measure their blood sample levels. Informed written consent was obtained from patients and family members.

### Study participants

One hundred nine clinically suspected CADASIL participants with NOTCH3 genetic testing between July 2013 and April 2019 were recruited. All the patients presented with one or more of the following manifestations [9]: 1) ischemic episodes, 2) cognitive deficits, 3) migraine with aura, 4) psychiatric disturbances, and 5) acute reversible encephalopathy. Additionally, all the patients presented with radiologic features of CSVD, including lacunar infarctions and WMHs [21]. WMHs were defined as hyperintense on T2-weighted sequences and isointense or hypointense (although not as hypointense as CSF) on T1-weighted sequences. A lacune is defined as a round or ovoid lesion ≥3 mm and ≤15 mm in diameter found on T1-weighted and T2-weighted images with a perilesional halo on FLAIR images [22, 23].

**Figure 1.**
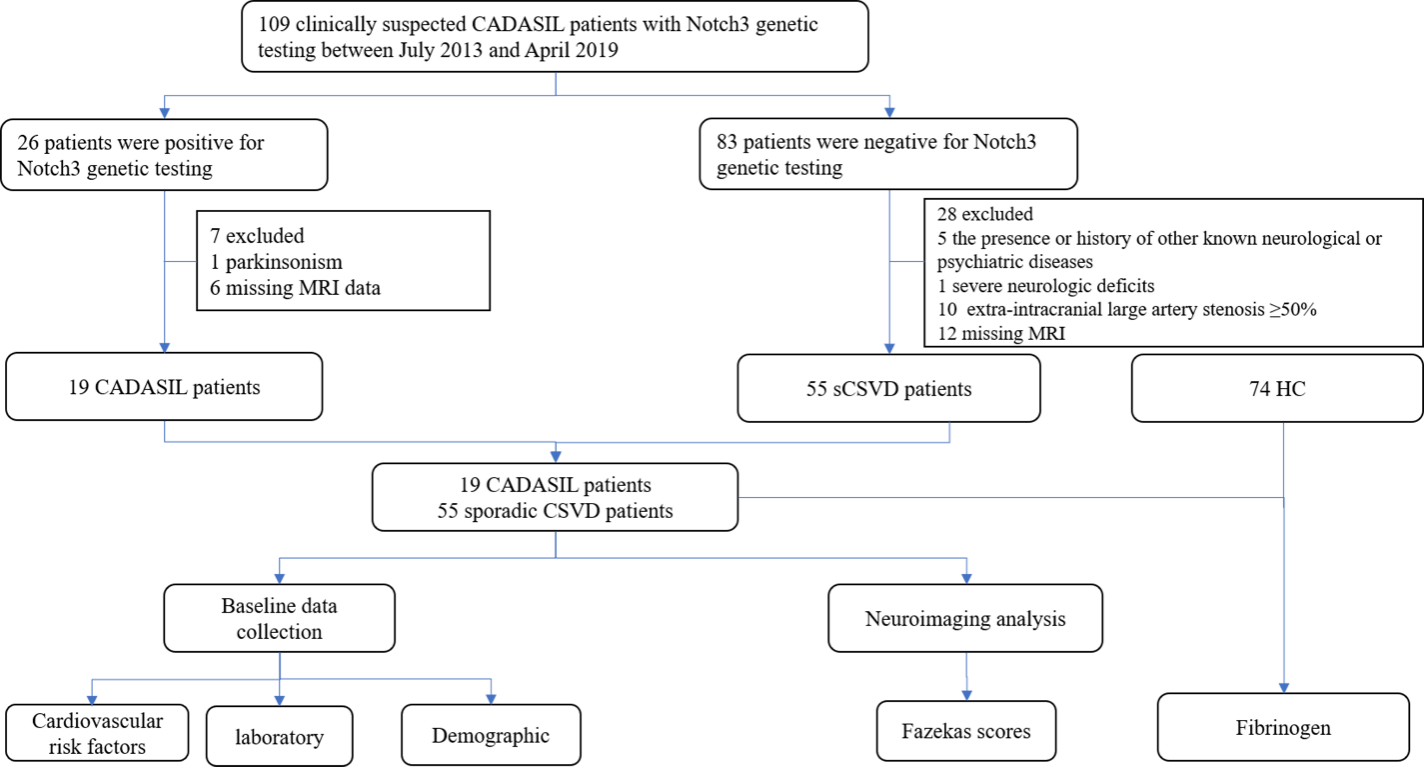
Study flow chart. One hundred nine clinically suspected CADASIL patients with Notch3 genetic testing between July 2013 and April 2019 were enrolled in this study. Thirty-five patients met the exclusion criteria. Finally, 19 CADASIL patients and 55 sCSVD patients were enrolled in this study. For further analysis, 66 healthy controls (HCs) were enrolled from the physical examination center. The plasma levels of fibrinogen were compared among the CADASIL, sCSVD and HC groups. Abbreviations: CADASIL, Cerebral Autosomal-Dominant Arteriopathy with Subcortical Infarcts and Leukoencephalopathy; sCSVD, sporadic cerebral small vessel disease; HC, health control.

The study flow diagram is shown in Figure 1. Of the 109 CSVD patients, 27 were positive for NOTCH3 genetic testing and diagnosed with CADASIL. One CADASIL patient was excluded because of the presence of parkinsonism, and 6 CADASIL patients were excluded because of missing MRI data at our hospital. Eighty-three patients were negative for NOTCH3 genetic testing and were diagnosed with sporadic CSVD (sCSVD). We excluded 12 sCSVD patients who had missing MRI data at our hospital and 10 patients with extra-intracranial large-artery stenosis ≥50%. Others with viral encephalitis (n=1), Parkinson’s disease (n=2), Hashimoto encephalopathy (n=1), multiple sclerosis (n=1), and severe neurologic deficits (n=1), were excluded from this study. Thus, the final analysis was performed in 19 CADASIL patients and 55 sporadic CSVD patients. Additionally, 74 age- and gender-matched healthy controls (HCs) were evaluated from the physical examination center at Zhujiang Hospital. The absence of cerebrovascular events or other neurological symptoms/diagnoses was established through a clinical interview and neurological examination by two neurologists. Written and informed consent was obtained from all the subjects after receiving a complete description of the study.

### Clinical characterization

Eligible participant characteristics that were regarded as likely relevant confounders of WMHs or fibrinogen were recorded as the baseline data. In this study, the following variables were collected: 1) demographic data (gender, age and BMI); 2) vascular risk factors (hypertension, diabetes, prior transient ischemic attack (TIA)/stroke, coronary heart disease (CHD), and history of smoking and drinking), with hypertension and diabetes mellitus defined as a self-reported medical diagnosis, antihypertensive/antidiabetic medication use, or a new diagnosis according to increased fasting and postprandial blood glucose; 3) laboratory markers such as cholesterol metabolism-relevant biomarkers (including LDL-cholesterol (LDL-C), HDL-cholesterol (HDL-C), total cholesterol (TC) and triglyceride (TG)) kidney function-relevant biomarkers (including creatinine (Cr) and uric acid (UA)); inflammatory markers such as the erythrocyte sedimentation rate (ESR), hypersensitive C-reactive protein (hs-CRP), leukocyte and neutrophil granulocyte (NEU) levels and the fibrinogen level.

### Laboratory biomarker measurements

Blood was collected between 8:30 and 10:30 a.m. after an overnight fast. Venous blood was collected in tubes that contained 10% by volume of 0.13 mol/L of sodium citrate. Plasma was isolated within 2 h by centrifugation at 2000 g at 4°C for 20 min and was stored at -80°C before assay. The plasma fibrinogen levels were examined using commercial kits following the manufacturer's instructions and a coagulometer (SC40 semi-automatic coagulation analyzer; Taizhou Steellex Biotechnology Co., Ltd., China). The levels of TC, LDL-C, HDL-C, TG, Cr, UA, ESR, leukocytes, hs-CRP and NEU were also analyzed and recorded by doctors who were blinded to the clinical and nervous system data of the patients.

### MRI scanning and analysis

In this study, all the patients were examined using a 3-T MR scanner (Magneton Tim Trio; Siemens, Erlangen, Germany) with a 32-channel head coil and a standardized protocol including T1, fluid-attenuated inversion recovery, and T2 sequences. WMHs were defined as the hyperintensity detected on both fluid-attenuated inversion recovery and T2-weighted images, without prominent hypointensity on T1-weighted images[23]. The severity of white matter hyperintensities (WMHs) was rated according to the Fazekas scale by two raters (Kappa=0.84). According to the Fazekas scores, the CSVD patients were divided into three groups (1: mild WMHs, 2: modest WMHs, 3, severe WMHs) [24].

### Statistical analysis

Statistical analyses were performed in ‘R,’ version 3.6.1. A p-value < 0.05 was considered significant. All the continuous variables—for example, age, TC, LDL-C, HDL-C, TG, ESR, Cr, UA, ESR, leukocyte level, NEU, hs-CRP and fibrinogen level—were shown as means ± SD; all categorical variables—such as gender, hypertension, diabetes, prior TIA/Stroke, CHD, and history of smoking and drinking, were presented as percentages. The Fazekas scores were shown as medians (interquartile range, IQR).

Statistically significant differences between CADASIL and sCSVD patients were assessed by t-test, χ^2^-test or the Mann-Whitney U test. P-values<0.05 were considered statistically significant. All categorical variables were assessed by χ^2^-test. Hs-CRP were assessed by Mann-Whitney U test as it was not normally distributed. other continuous variables which are normally distributed were assessed by t-test. Fazekas scores and Modified Rankin Scale (MRS) scores were also assessed by Mann-Whitney U test. Mini-mental State Examination (MMSE) scores were assessed by t-test. To compare the difference in the fibrinogen levels among CADASIL patients, sporadic CSVD patients and healthy control (HCs), we used the Welch test (with Dunnett’s T3 test for multiple comparisons) because of the heterogeneity of variance between groups. The ability of plasma fibrinogen to discriminate between CADASIL or sporadic CSVD patients and HCs was assessed using receiver operating characteristic (ROC) analysis as implemented in the R package “ROCR” [25]. Statistically significant differences between patients with different severities of WMHs were assessed by one-way analysis of variance (one-way ANOVA), χ^2^-test or the Kruskal-Wallis test. When multiple testing was performed, the Bonferroni method was used to adjust the significance level. All categorical variables were assessed by χ^2^-test. Hs-CRP and ESR were assessed by Kruskal-Wallis test as they were not normally distributed. other continuous variables which are normally distributed were assessed by one-way ANOVA.

Multivariate ordinary logistic analysis was performed to detect the significantly independent associated factors for the severity of WMHs in CSVD. In regression model, variables for inclusion were carefully chosen, given the number of samples, to ensure parsimony of the final model. The baseline variables that were considered clinically relevant (including age, hypertension and smoking) [26] or that showed univariate relevance with the severity of WMHs (P<0.1) were entered into the multivariate regression model. P-values<0.05 were considered statistically significant.

**Table 1:** Characteristics of the patients with Notch3 genetic testing.

Characteristic	CADASIL (n=19)	sCSVD (n=55)	t,χ2 or Z	P-Value
Demographic characteristics				
Age (yr), mean±SD	56.9±11.6	55.7±13.7	0.352	0.726
Female sex, n (%)	5(26.3)	13(23.7)	0.055	0.814
BMI (kg/m2), mean±SD	23.86±3.56	23.46±2.92	1.005	0.660
Vascular risk factors				
Hypertension, n (%)	8 (42.1)	38 (69.1)	4.278	0.039
Diabetes, n (%)	0 (0)	8 (14.5)	1.744	0.183
History of smoking, n (%)	4 (21.1)	15 (27.2)	0.484	0.593
History of drinking, n (%)	4(21.1)	5 (9.1)	0.034	0.169
Prior TIA/Stroke, n (%)	15(78.9)	45(81.8)	0.076	0.783
CHD, (n%)	0(0)	5(9.1)	1.852	0.319
Clinical scores				
WMHs, median (IQR)	3 (1)	2 (1)	-2.126	0.033*
MRS, median (IQR)	2 (1)	1 (1)	-0.879	0.379
MMSE, mean±SD	22.4±7.4	23.1±5.6	-1.665	0.100
Laboratory				
Fibrinogen (mg/dL), mean±SD	362.63±91.06	358.65±92.37	0.122	0.904
TC (mmol/L), mean±SD	4.14±0.80	4.16±1.25	1.094	0.277
LDL-C (mmol/L), mean±SD	2.32±0.86	2.41±0.86	0.431	0.718
HDL-C (mmol/L), mean±SD	1.34±0.45	1.09±0.32	2.785	0.007*
TG (mmol/L), mean±SD	1.20±0.46	1.66±1.04	-2.208	0.046*
Cr (μmol/L), mean±SD	77.12±24.15	85.7±29.2	-1.157	0.251
UA (μmol/L), mean±SD	344.52±66.05	357.5±109.6	-0.450	0.573
ESR (mm/h), mean±SD	11.15±6.89	23.35±9.71	-3.941	0.001*
leukocyte (*10^9^/L), mean±SD	6.90±1.08	7.22±1.66	-0.948	0.348
NEU (%), mean±SD	56.43±15.58	57.24±14.80	-0.215	0.830
Hs-CRP (mg/L), mean±SD	2.76±5.84	2.18±3.22	-0.689	0.491

Interquartile range (IQR) is defined as the difference between the 75th and 25th percentiles. CADASIL, Cerebral autosomal dominant arteriopathy with subcortical infarcts and leukoencephalopathy; sCSVD, sporadic cerebral small vessel disease; TIA, transient ischemic attack; WMHs, white matter hyperintensities; LDL-C , LDL-cholesterol ; HDL-C ,HDL-cholesterol ; TC , total cholesterol; TG ,Triglyceride ; Cr, creatinine ; UA , Uric acid; ESR ,erythrocyte sedimentation rate ; NEU , neutrophil granulocyte, Hs-CRP, hypersensitive C-reactive protein; CHD, coronary heart disease; MMSE, Mini-mental State Examination; MRS, Modified Rankin Scale. *P<0.05. The statistically significant differences between CADASIL and sCSVD patients were assessed by the t-test, χ2-test or Mann-Whitney U tests. P-values<0.05 were considered statistically significant.

## RESULTS

### Participant characteristics

Seventy-four participants (19 CADASIL patients and 55 sCSVD patients) were successfully screened and enrolled in the study. The patients’ characteristics, including sex, age, BMI, medical history (hypertension, CHD and diabetes), history of drinking and smoking, fibrinogen level, and biomarkers of systemic inflammation (leukocyte, NEU, ESR and hs-CRP) were summarized and included in Table 1. Interestingly, the severities of WMHs and HDL-C in CADASIL patients were significantly higher than those in sCSVD patients. Additionally, the ESR in CADASIL patients was significantly lower than that in sCSVD patients. No significant differences were found in age, BMI, cholesterol, LDL-C, leukocyte level, hs-CRP, neutrophil granulocyte level, Cr, UA, sex, hypertension, history of smoking and drinking, diabetes, CHD, MMSE scores and MRS scores between the groups in this study.

**Figure 2.**
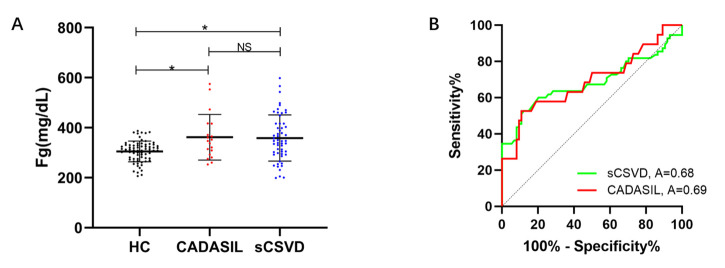
increased plasma levels of fibrinogen in CADASIL and sporadic CSVD patients. (A) Compared with health controls (HCs), the plasma levels of fibrinogen were increased in CADASIL and sCSVD patients. (B) Receiver operating characteristic analyses for plasma fibrinogen: CADASIL patients vs. HCs (red line) and sCSVD patients vs. HC (green line). The AUC was 0.69 for CADASIL and 0.68 for sCSVD patients. Abbreviation: HC, health control; CADASIL, Cerebral Autosomal-Dominant Arteriopathy with Subcortical Infarcts and Leukoencephalopathy; sCSVD, sporadic cerebral small vessel disease; AUC, area under the curve; Fg, fibrinogen. *compared to HC, P<0.05.

### Comparison of the plasma fibrinogen levels in CADASIL, sCSVD and HC patients

Compared with healthy controls, the plasma fibrinogen levels were significantly higher in both CADASIL patients (P=0.046) and sporadic CSVD patients (P<0.001) but showed no difference between the CADASIL and sCSVD patients (P=0.904) (Fig. 2A). The AUCs for fibrinogen to discriminate between HC and CADASIL or sCSVD patients were 0.69 and 0.68, as shown in Figure 2B.

### Univariate analysis for possible factors associated with the severity of WMHs

**Figure 3.**
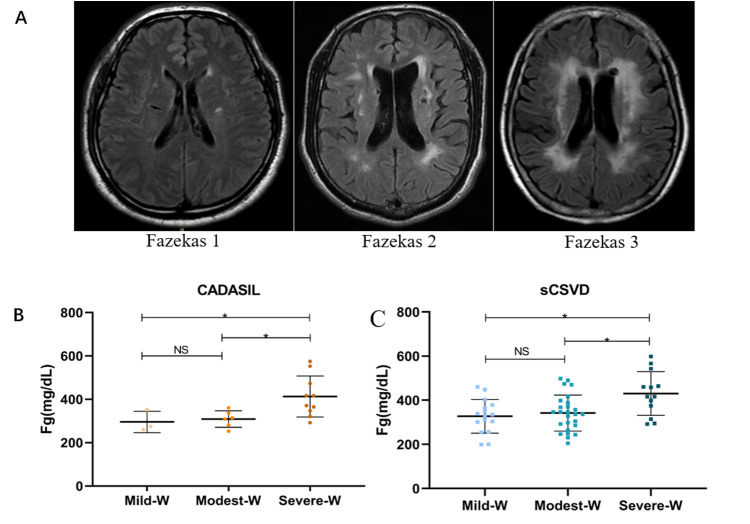
Comparison of the plasma levels of fibrinogen in CADASIL and sCSVD patients with different severities of WMHs. (A) Representative MRI imaging (T2 FLAIR) of WMHs in CSVD patients with different severities classified by the Fazekas score. In both CADASIL. (B) and sCSVD (C) samples, the plasma levels of fibrinogen were relatively higher in patients of severe-W (Fazekas score =3) than in those with mild-W (Fazekas score=1) or modest-W (Fazekas score=2). Abbreviation: CADASIL, Cerebral Autosomal-Dominant Arteriopathy with Subcortical Infarcts and Leukoencephalopathy; sCSVD, sporadic cerebral small vessel disease; Fg, fibrinogen; mild-W, mild white matter hyperintensities; modest-W, modest white matter hyperintensities; severe-W, severe white matter hyperintensities; FLAIR, fluid attenuated inversion recovery. *Compared to moderate-W or mild-W, P<0.05.

According to the Fazekas scores, the CSVD patients were divided into three groups according to the severity of WMHs (1: mild WMHs; 2: modest WMHs; 3, severe WMHs), as shown in Figure 3A. The possible factors associated with the severity of WMHs in CADASIL and sCSVD patients are summarized in Table 2. We found that both CADASIL and sCSVD patients with severe WMHs exhibited higher plasma levels of fibrinogen than cases with mild or modest WMHs (Fig. 3B). In sCSVD patients, cases with modest or severe WMHs were older than patients with mild WMHs, but no significant differences in age were noted in CADASIL patients with different grades of WMHs. Additionally, patients with severe WMHs exhibited higher levels of hs-CRP in CADASIL but not in sCSVD cases. No significant difference was found in BMI, sex, TC, LDL-C, LDL-C, TG, Cr, UA, ESR, leukocyte level, NEU, prior TIA/stroke, hypertension, diabetes, CHD, and history of smoking and drinking among patients (both CADASIL and sCSVD) with different severities of WMHs.

### Multivariable logistic regression analysis of risk factors of the severity of WMHs

**Figure 4.**
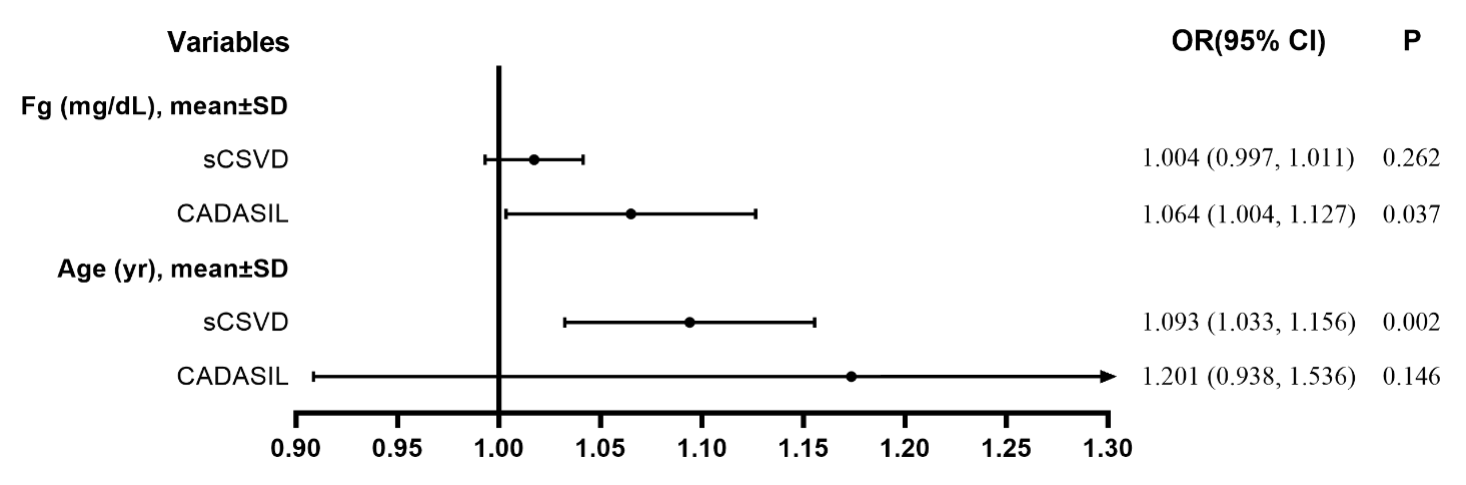
Multivariate analysis of independent variables associated with the severity of WMHs. Abbreviations: CADASIL, Cerebral Autosomal-Dominant Arteriopathy with Subcortical Infarcts and Leukoencephalopathy; sCSVD, sporadic cerebral small vessel disease; WMHs, white matter hyperintensities.

Ordinal logistic regression models were used to examine the association between fibrinogen and the severity of WMHs. In CADASIL patients, a higher fibrinogen level, but not increased age, was an independent risk factor associated with the severity of WMHs. However, increased age, but not a higher fibrinogen level, was an independent risk factor for the severity of WMHs in sCSVD patients (Table 3 and Fig. 4).

## DISCUSSION

CSVD is the leading vascular cause of cognitive decline and dementia, which common imaging feature is WMHs on MRI. However, risk factors of WMHs are multifactorial and still more controversial. Fibrinogen is an inflammatory and systemic hypercoagulability marker. High levels of fibrinogen in plasma might reduce blood flow, predispose to thrombosis, and enhance atherogenesis [27]. Some population-based studies investigated the role of fibrinogen in asymptomatic CSVD patients, but very few studies investigated the relationships between WMHs and fibrinogen in disparate subtypes of CSVD. In this study, we first explored the relationship between fibrinogen and WMHs in different CSVD subtypes, including CADASIL and sCSVD patients. We noted several interesting findings. First, the grades of WMHs in CADASIL patients were significantly higher than those in sCSVD patients;Second, both CADASIL and sCSVD patients showed significantly higher plasma fibrinogen levels than HCs. Third, both CADASIL and sCSVD patients with severe WMHs exhibited higher plasma levels of fibrinogen than patients with mild or modest WMHs. Finally, a higher fibrinogen level, but not increased age, was found to be an independent risk factor associated with the severity of WMHs in CADASIL patients but not in sCSVD patients.

**Table 2:** Univariate analysis of possible variables associated with severity of WMHs.

Risk factors	Mild-W (n=16)	sCSVD			CADASIL			

		Modest-W (n=26)	Severe-W (n=13)	P	Mild-W (n=3)	Modest-W (n=6)	Severe-W (n=10)	P
Age (yr), mean±SD	45.06±10.85	57.89±13.05	64.46±10.05	<0.001*	53.33±7.09	56.33±11.52	58.40±13.09	0.811
BMI (kg/m^2^), mean±SD	23.44±2.03	23.20±3.17	23.98±3.45	0.743	23.13±4.62	25.80±3.21	23.21±3.37	0.256
Female sex, n (%)	4(25.0)	8(30.8)	1(7.7)	0.306	0(0.0)	2(33.3)	3(30.0)	0.640
Fibrinogen (g/L), mean±SD	327.44±76.30	342.00±81.53	430.38±98.95	0.004*	295.67±49.36	309.00±38.29	413.00±94.87	0.023*
TC (mmol/L), mean±SD	3.87±0.95	3.95±0.75	3.80±1.12	0.554	4.42±0.32	4.13±0.96	4.06±0.84	0.817
LDL-C (mmol/L), mean±SD	2.29±0.61	2.58±1.01	2.22±0.78	0.386	2.98±0.36	2.50±0.79	2.02±0.93	0.213
HDL-C (mmol/L), mean±SD	1.05±0.30	1.13±0.36	1.02±0.24	0.570	1.15±0.14	1.26±0.33	1.45±0.56	0.549
TG (mmol/L), mean±SD	1.80±0.87	1.49±0.81	1.74±1.33	0.442	1.19±0.45	1.45±0.24	1.00±0.53	0.182
Cr (μmol/L), mean±SD	81.91±22.43	79.68±19.81	102.62±44.53	0.054	74.40±16.91	80.46±16.40	75.93±30.69	0.924
UA (μmol/L), mean±SD	318.19±74.46	366.32±121.47	384.08±113.71	0.225	347.73±102.56	332.45±77.85	350.80±53.63	0.875
ESR (mm/h), mean±SD	19.81±12.78	21.12±18.66	32.15±26.59	0.348	8.00±6.08	7.33±4.97	14.40±6.95	0.083#
Hs-CRP (mg/L), mean±SD	2.11±2.00	1.64±2.68	3.35±4.96	0.126	0.50±0.00	0.82±0.74	4.61±7.74	0.033*
leukocyte (*10^9^/L), mean±SD	7.34±1.82	6.94±1.61	7.60± 1.61	0.480	6.36±1.23	6.74±0.29	7.16±1.32	0.509
NEU (%), mean±SD	58.67±10.64	59.14±9.98	58.50±9.83	0.817	56.10±8.23	53.45±7.85	62.53±10.07	0.173
Prior TIA/Stroke, n (%)	13(81.3)	21(80.7)	11(84.6)	0.999	3(100.0)	5(83.3)	7(70.0)	0.791
Hypertension, n (%)	9(56.3)	19(73.1)	11(84.6)	0.256	0(0.0)	4(66.6)	6(60.0)	0.187
Diabetes, n (%)	2(12.5)	5(19.2)	1(7.7)	0.702	0(0.0)	0(0.0)	0(0.0)	-
CHD, (n%)	1(6.3)	1(3.8)	3(23.1)	0.133	0(0.0)	0(0.0)	0(0.0)	-
History of smoking, n (%)	3(18.8)	7(26.9)	4(30.8)	0.786	1(33.3)	1(16.6)	4(40.0)	0.801
History of drinking, n (%)	2(25.0)	1(3.8)	3(23.1)	0.175	1(33.3)	1(16.6)	3(30.0)	0.999

CADASIL, Cerebral autosomal dominant arteriopathy with subcortical infarcts and leukoencephalopathy; CSVD, cerebral small vessel disease; TIA, transient ischemic attack; WMHs, white matter hyperintensities; LDL-C, LDL-cholesterol; HDL-C, HDL-cholesterol ; TC , total cholesterol; TG ,Triglyceride ; Cr, creatinine ; UA , Uric acid; ESR ,erythrocyte sedimentation rate ; NEU , leukocyte and neutrophil granulocyte, Hs-CRP, hypersensitive C-reactive protein; coronary heart disease. *P<0.05, #P<0.1. The statistically significant differences between patients with different severity of WMHs were assessed by the one-way analysis of variance, χ2-test or Kruskal-Wallis test.

Few studies compared the grade of WMHs between CADASIL and sCSVD. This result may be explained by the mediation effect of lacunar infarcts [28]. In our cohort, we found that presence of lacunes were higher in CADASIL than sCSVD (100% vs 78.2%, P=0.029). Lacunes may promote formation of WMHs, possibly through a?ecting white matter tract integrity [29]. Fibrinogen is a crucial role in coagulation cascade and inflammation. Beamer et al [30] found that fibrinogen levels were elevated in stroke survivors compared with those in healthy control groups even 1 year after stroke onset, which were consistent with our study. It is widely believed that fibrinogen is involved in the pathogenesis of chronic ischemia of white matter, inflammatory cascade activation, blood-brain barrier (BBB) leakage caused by CSVD [31]. This may explain the higher level of plasma fibrinogen in CSVD than HCs.

The findings regarding the relationship between fibrinogen and WMH are inconsistent. Two large general population-based cohort studies have shown that the fibrinogen levels were associated with the presence of WMH [32, 33]. In a prospective study, the evaluated fibrinogen level increased the risk of WMH progression [34]. However, two other large population-based studies found that the presence of WMHs was not associated with the presence of WMHs [35, 36]. Another sizeable population-based study found that the WMH severity was not associated with the fibrinogen levels [37]. In the current study, associations between the fibrinogen levels and WMHs remained statistically significant in CADASIL but not in sCSVD patients even after adjustment for various confounding variables. This finding indicated that the relationships between WMHs and fibrinogen in disparate subtypes of CSVD were different. In univariate analysis, both CADASIL and sCSVD patients with severe WMHs exhibited higher plasma levels of fibrinogen than patients with mild or modest WMHs. However, the associations between fibrinogen and WMHs were not significant in sCSVD patients after the adjustment for age. The discrepancy between CADASIL and sCSVD patients may be explained by the fibrinogen levels increasing with age in sCSVD but not in CADASIL patients. Besides, in CADASIL, the underlying vascular lesion is a specific non-atherosclerotic, amyloid-negative angiopathy involving small arteries and capillaries, primarily in the brain [38], which could explain the different association between CADASIL and sCSVD in pathological perspective.

**Table 3:** Multiple ordinal logistic regression with severity of WMH as dependent variables.

variables	sCSVD		CADASIL	
	OR (95%Cl)	P	OR (95%Cl)	P
Age (yr), mean±SD	1.093(1.033,1.156)	0.002*	1.154 (0.919, 1.448)	0.218
Fibrinogen (mg/dL), mean±SD	1.004(0.997,1.011)	0.262	1.064 (1.004, 1.127)	0.037*
Hypertension, n (%)	1.263(0.341,4.675)	0.726	6.340(0.156, 257.509)	0.357
History of smoking, n (%)	2.714(0.580,8.155)	0.250	1.230 (0.067, 22.437)	0.889
Cr (μmol/L), mean±SD	1.024(1.000,1.048)	0.054	-	-
Hs-CRP (mg/L), mean±SD	-	-	1.367(0.100, 18.761)	0.815
ESR (mm/h), mean±SD	-	-	1.224(0.943, 1.641)	0.123

Multivariate ordinary logistic analysis was performed to detect the significantly independent associated factors for severity of WMHs in CSVD. In the model of sCSVD patients, all confounders were enrolled. In model of CADASIL patients, variables for inclusion were carefully chosen, given the number of samples, to ensure parsimony of the final model. Baseline variables that were considered clinically relevant (including age, hypertension and smoking) or that showed a univariate relevant with severity of WMHs (P<0.1) were entered into the multivariate regression model. P-values<0.05 were considered statistically significant. CADASIL, Cerebral autosomal dominant arteriopathy with subcortical infarcts and leukoencephalopathy; sCSVD, sporadic cerebral small vessel disease; TIA, transient ischemic attack; WMHs, white matter hyperintensities; LDL-C , LDL-cholesterol ; HDL-C ,HDL-cholesterol ; TC , total cholesterol; TG ,Triglyceride ; Cr, creatinine ; UA , Uric acid; ESR ,erythrocyte sedimentation rate ; NEU , neutrophil granulocyte, Hs-CRP, hypersensitive C-reactive protein; CHD, coronary heart disease . *P<0.05.

The exact mechanism by which elevated fibrinogen might contribute to WMHs remains unknown. However, WMHs are considered to reflect ischemic small vessel disease. Fibrinogen triggers various atherogenic processes such as endothelial injures [39]. Thus, fibrinogen might promote atherogenesis in small vessels. Elevated fibrinogen levels induce hypercoagulability and might reflect the progression of atherosclerosis. Such hemorheological impairments caused by increased levels of fibrinogen would aggravate cerebral hypoperfusion [40]. Hyperfibrinogenemia could be alleviated by making lifestyle modifications or drug usage. Higher intakes of iron, sugar, and caffeine, in addition to obesity, mainly account for higher fibrinogen levels [41]. Several drugs could reduce fibrinogen levels, including bezafibrate and ticlopidine[42]. However, insufficient evidence supports the routine use of fibrinogen-lowering agents to delay the progression of WMHs. Further study is needed to clarify this issue.

Several limitations should be noted. First, we have not evaluated other CSVD neuroimaging markers, such as the mean diffusivity. Particular sequences, such as susceptibility-weighted imaging and diffusion-weighted imaging, should be conducted to evaluate the CSVD burden. Second, only semi-quantitative method was used to identify the severity of WMHs in our study. The methods of WMH quantification (Semi-quantitative and quantitative) in different studies were compared; theoretically, it might be easier to identify the association between WMHs and fibrinogen by quantitative methods. However, recent studies using quantitative methods didn’t show the association between fibrinogen and WMH [35, 43]. Furthermore, the results obtained on the scale are correlated closely with the volumetric assessments [44]. This confounder may have minimal impact on comparing different studies. Third, the current study is limited mainly by its retrospective nature, assessing the plasma levels of fibrinogen in a relatively small sample size of sCSVD and CADASIL patients at a single time point. Future prospective and longitudinal studies are needed, while randomized trials of drugs that can reduce plasma fibrinogen may help clarify whether the decrease in fibrinogen could delay the progression of WMHs in CSVD patients.

In conclusion, the present study demonstrated that plasma fibrinogen levels are independently associated with the severity of WMHs in CADASIL patients but not in sCSVD patients, suggesting that the role of fibrinogen may be different in disparate subtypes of CSVD.
